# Alcohol and Substance Use in the Jewish Community: A Pilot Study

**DOI:** 10.1155/2015/763930

**Published:** 2015-06-16

**Authors:** Melanie Baruch, Abraham Benarroch, Gary E. Rockman

**Affiliations:** ^1^University of Manitoba, Winnipeg, MB, Canada; ^2^Jewish Child and Family Service, Winnipeg, MB, Canada; ^3^Department of Psychology, University of Winnipeg, 515 Portage Avenue, Winnipeg, MB, Canada R3B 2E9

## Abstract

Awareness of addictions in the Jewish community is becoming increasingly prevalent, and yet, a gap exists in the literature regarding addictions in this community. Knowledge about the prevalence of addictions within Jewish communities is limited; some believe that Jews cannot be affected by addictions. To address this gap, a pilot study was conducted to gather preliminary evidence relating to addictions and substance use in the Jewish community. Results indicate that a significant portion of the Jewish community knows someone affected by an addiction and that over 20% have a family history of addiction. Future research needs are discussed.

## 1. Introduction

Approximately 11% of the Canadian population struggles with substance use [1]. The prevalence of alcohol and substance use in the Jewish community remains uncertain, possibly due to the existence of some denial of addictive behaviours in this community [2]. Israeli evidence documenting the lifetime prevalence of drug use in Israel is 13% (as cited in [2]). Yet, to date, it appears that a large portion of the North American Jewish community views alcoholism as an illness, has a strong fear of alcoholics, and blames individuals with addictions for their condition [3, 4]. One possible conclusion is that Jewish people believe that members of the Jewish community simply do not become alcoholics, so they are convinced that they are not exposed to people with addictions [5]. Therefore, they lack the ambition to seek education on the topic and become naive to the reality of the prevalence of addictions in the Jewish community [5]. Glass [6] discusses the myth existent across Jewish communities that Jews cannot have addictions. “Over the years, this long legacy of denial among Jews has resulted in unnecessary pain, heartache, and a great deal of alienation from Judaism by those suffering from addiction. It has also served to prevent some suffering Jews from seeking or accepting appropriate help” [6, page 235]. The refusal of Jewish alcoholics is also shown when a large number of Jewish people claim that they do not know any heavy drinkers [3]. These views contribute to an active denial stage in Jewish addicts [5]. The belief that Jews do not become alcoholics results in leaders of the community failing to address the problem and discourages health professionals to conclude the diagnosis of an addiction of a Jewish person [5]. While these views may have changed on a societal level, empirical evidence on this topic remains limited, resulting in outdated reflections of addictions in Jewish populations which may not accurately represent the current reality. For instance, Jewish Alcoholics, Chemically Dependent Persons, and Significant Others, commonly known as JACS, is a self-help program for Jews and loved ones coping with addictive behaviours [6]. JACS groups are located throughout Canada, the United States, Australia, Brazil, and Israel [7] and are indicative of the existence of addictions in Jewish communities internationally.

Jewish individuals are likely to be impacted by addictions, similar to other ethnicities [8]. However, due to the stereotype that Jews cannot have addictions [5, 9], Jews affected by addictions may find themselves with limited supports within their own communities [5]. The purpose of this study is to provide preliminary evidence relating to addictions and substance use in the Jewish community.

## 2. Methods

A survey was administered to adults receiving services through Jewish Child and Family Service, Winnipeg location. A package was mailed to randomly selected individuals, where they were asked to fill out the questionnaire and mail it back to the Jewish Child and Family Service office. The package mailed to participants included a guide detailing the contents of the package, a consent form, a questionnaire, a feedback form, a Jewish Alcoholics, Chemically Dependent Persons, and Significant Others (JACS, a Jewish 12-step group) bookmark, and a stamped envelope to mail the package back once completed. Participants were contacted two weeks after packages had been sent in order to inform them that they have been randomly selected to participate in the study and to provide an opportunity to ask questions and express concerns.

*Sample.* Participants consisted of adult men and women receiving services from Jewish Child and Family Service (JCFS). Participants were randomly selected from every service area at Jewish Child and Family Service. Service areas included newcomer settlement services, mental health, child welfare, adoption, and counselling. Except for counselling clients, all other JCFS clients must be Jewish to receive services from JCFS.

*Survey Instruments.* Participants completed a 7-page questionnaire that examined participants' drug and alcohol use. The questionnaire is heavily based on questions from 4 addictions measures including the South Oaks Gambling Screen (SOGS), Brief Michigan Alcoholism Screening Test (BMAST), and the Drug Abuse Screening Test (DAST).

The SOGS was developed in 1987 by Dr. Henry Lesieur and Dr. Sheila Blume for the purpose of identifying pathological gambling [10]. The present study included 4 items from the SOGS (“Have you ever gambled more than you intended to? Do you feel you have ever had a problem with gambling? Have you ever felt guilty about the way you gamble or what happens when you gamble? Have you ever borrowed money from someone to gamble or to pay gambling debts?”). The BMAST is a ten-item questionnaire widely used to assess alcohol dependence. The BMAST stems from the 25-item Michigan Alcoholism Screening Test (MAST) [11]. Specifically, the BMAST assesses alcohol consumption, drinking behaviour and dependence, and consequences of drinking [11]. The DAST is a 28-item questionnaire developed by Dr. Harvey A. Skinner [12]. 13 representative items from the DAST were incorporated into the survey instrument.

## 3. Results

The present study had an 11.5% response rate (*N* = 295, *n* = 34), which is lower than average response rate for mailed questionnaires [13], however, an acceptable sample size for a pilot study [14]. The results of analyses of correlations among income, missing work as a result of drug use, and fighting under the influence of drugs are listed in Table 1.

The majority of respondents were female (84.4%) and nearly half (41.2%) were over 61 years old. The majority of respondents' education ranged from some university education to graduate degrees. 82.4% of respondents identified their religious affiliation as Jewish, 8.8% Catholic, 2.9% Protestant, and 2.9% Atheist. An analysis of participants by service area is provided in Table 2.

41.2% reported knowing someone currently struggling with an addiction, and 23.5% of respondents reported having a family history of alcohol or drug abuse. When asked if participants or loved ones required help with substance abuse or gambling, 8.8% stated they would contact a rabbi or priest, 11.8% a CFS agency, 8.8% their doctor, 2.9% AA, 2.9% AFM, and 5.9% private counselling, and 2.9% would not seek help. 44.1% of respondents stated they would seek out multiple community resources including Alcohol Anonymous (66.6%), the Addictions Foundation of Manitoba (73.3%), private counselling (53.3%), Child and Family Service Agency (46.6%), Employee Assistance Program (20%), inpatient facilities (20%), and a rabbi or priest (20%). 35.3% of respondents reported knowing that JCFS offers JACS and 70.6% stated they would consider attending a JACS meeting. Knowing someone currently struggling with an addiction was positively correlated with knowledge that JCFS offers JACS (*r* = .358, *p* = .044). Additionally, marital status positively correlated with knowledge of addictions services at JCFS (*r* = .423, *p* = .016).

17.6% of respondents stated they have used drugs other than those required by medical reasons. 14.7% of these participants stated they cannot get through the day without using drugs and 2.9% reported neglecting their family because of their drug use. 8.8% sought help for drug use. Sex, age, marital status, and education were individually examined as predictors of alcohol and substance use; all were nonsignificant (*p*s > .05).

A linear regression analysis showed that knowing someone currently struggling with an addiction explains 12.8% of the variability in knowing that JCFS offers a JACS support group; *F*(1,30) = 4.405; *p* < .05. Furthermore, marital status accounts for 17.9% of the variance in knowing that JCFS offers a JACS support group; *F*(1,30) = 6.556; *p* < .05.

## 4. Discussion

Research remains in its infancy on whether Jewish people feel that addictions in the Jewish community are nonexistent and therefore are not educated on the topic [5]. The current study extends this knowledge base by providing preliminary insight into the awareness of the Jewish community on others struggling with addictions, as well as services available. 41.2% reported knowing someone currently struggling with an addiction, and 23.5% of respondents reported having a family history of alcohol or drug abuse. These statistics speak to the need to further explore the Jewish community's role in recovery within the Jewish community. A statistically significant positive relationship between knowing someone struggling with an addiction and familiarity with JCFS's addictions support services is indicative of the fact that those directly affected are making an effort at identifying the resources available within their community. Marital status accounts for almost 18% of the variance in familiarity with JACS. While currently there is no research to justify these results, it is possible that concern over a spouse's behaviour as it relates to alcohol or substance use may result in increasing one's familiarity with supportive services. Significant positive relationships between income and alcohol and substance use need to be further investigated.

The present findings must be considered with regard to limitations. This study did not target a random sample and used a small subset of the Jewish community already connected with a family service agency, failing to represent the entire Jewish community. Additionally, the low response rate could be indicative of the Jewish community's lack of desire to discuss and participate in conversation and research relating to addictions in the Jewish community. Older adults accounted for the greatest responding group, which may have influenced the results.

This study provides preliminary evidence for the increasing rates of addiction in the Jewish community and the need to develop awareness in addictions services in the Winnipeg Jewish Community. It indicates that further inquiry is necessary in the areas of addiction recovery in the Jewish community. Particularly, are Jewish addicts reaching out for help within the Jewish community, is religion or spirituality a significant factor in the recovery process, and are Jewish individuals with addictions feeling supported through recovery in the Jewish community?

## Figures and Tables

**Table 1:** Correlations between income and substance use.

	Income	Marijuana use	Alcohol use	Cocaine use	Prescription medication use	General recreational drugs	Fights under the influence of drugs	Miss work
Income		.413^*∗*^	.383^*∗*^	.419^*∗*^	.418^*∗*^	.250	.106	−.305
Marijuana use			.991^*∗∗*^	.998^*∗∗*^	.998^*∗∗*^	.693^*∗∗*^	.472^*∗∗*^	.307
Alcohol use				.991^*∗∗*^	.991^*∗∗*^	.673^*∗∗*^	.452^*∗∗*^	.296
Cocaine use					1.000^*∗∗*^	.696^*∗∗*^	.477^*∗∗*^	.314
Prescription medication use						.696^*∗∗*^	.476^*∗∗*^	.317
General recreational drugs							.685^*∗∗*^	.451^*∗∗*^
Fights under the influence of drugs								.658^*∗∗*^
Miss work								

Note: ^*∗*^ *p* < .05; ^*∗∗*^ *p* < .01.

**Table 2:** Participants by JCFS service area.

	Frequency	Percent
Adoption	1	2.9
Counselling	8	23.5
Newcomers	3	8.8
Older adults	11	32.4
Protection	1	2.9
Volunteers	3	8.8
Mental health	6	17.6
Unspecified	1	2.9
Total	34	100

## References

[1] Veldhuizen S., Urbanoski K., Cairney J. (2007). Geographical variation in the prevalence of problematic substance use in Canada. *Canadian Journal of Psychiatry*.

[2] Loewenthal K. M. (2014). Addiction: alcohol and substance abuse in Judaism. *Religions*.

[3] Glassner B., Berg B. (1984). Social locations and interpretations: how jews define alcoholism. *Journal of Studies on Alcohol*.

[4] Weiss S., Moore M. (1992). Perception of alcoholism among Jewish, Moslem and Christian teachers in Israel. *Journal of Drug Education*.

[5] Vex S. L., Blume S. B. (2001). The JACS study I: characteristics of a population of chemically dependent Jewish men and women. *Journal of Addictive Diseases*.

[6] Glass C., Morgan O. J., Jordan M. (1999). Addiction and recovery through Jewish eyes. *Addiction and Spirituality: A Multidisciplinary Approach*.

[7] The Jewish Board of Family and Children Services http://www.jbfcs.org/programs-services/jewish-community-services-2/jacs/meetings/#.VXDIMFxVikp.

[8] Engs R. C., Hanson D. J., Gliksman L., Smythe C. (1990). Influence of religion and culture on drinking behaviours: a test of hypotheses between Canada and the USA. *British Journal of Addiction*.

[9] Steiker L. H., Scarborough B. (2011). Judaism, alcoholism, and recovery: the experience of being jewish and alcoholic. *Journal of Social Work Practice in the Addictions*.

[10] Lesieur H. R., Blume S. B. (1993). Revising the south oaks gambling screen in different settings. *Journal of Gambling Studies*.

[11] Connor J. P., Grier M., Feeney G. F. X., Young R. M. (2007). The validity of the brief Michigan alcohol screening test (bMAST) as a problem drinking severity measure. *Journal of Studies on Alcohol and Drugs*.

[12] Yudko E., Lozhkina O., Fouts A. (2007). A comprehensive review of the psychometric properties of the drug abuse screening test. *Journal of Substance Abuse Treatment*.

[13] Tse A. (1998). Comparing the response rate, response speed and response quality of two methods of sending questionnaires: e-mail vs. mail. *Journal of the Market Research Society*.

[14] Hertzog M. A. (2008). Considerations in determining sample size for pilot studies. *Research in Nursing & Health*.

